# Acute Infantilism

**Published:** 1910-05

**Authors:** H. McH. Hull

**Affiliations:** Atlanta, Ga.


### ACUTE INFANTILISM.

BY H. MCH. HULL, M. D., ATLANTA, GA.

About two years ago, Dr. C. A. Herter, of New York, called attention to the existence of an affection which had previously been unrecognized—at least as far as the underlying causes, and some of the more obscure symptoms, were concerned. It is a condition occurring in childhood, the more prominent features of which are a retarding of growth physically, certain rhachitic symptoms, and the occurrence of frequent attacks of auto-intoxication, almost invariably following the ingestion of certain articles of diet, and associated with grayish, exceedingly offensive stools.

The mentality of the child is not affected. On the contrary, under ordinary circumstances, the child's mind seems to be more bright and active than normal, possibly because of comparison with other children of the same size, but not of the same age. To this condition he gave the name of acute infantilism; so designating it because of the existence in the intestinal tract of certain bacteria found in that of infants, and ordinarily not found in that of the other child, and called acute to designate it from the nervous disorder which ordinarily goes under the name of infantilism. This condition is primarily a disorder of the intestinal tract, and has nothing whatever to do with the other condition referred to above.

As pointed out by him in a very clear way, the prominent symptoms might be classified under the following heads:

Retarding of growth physically, associated with normal mental growth; frequent attacks of auto-intoxication, and marked indicanuria; an inability to digest starches and fats; the presence in the intestinal tract in large numbers of certain Gram poistive bacteria, more notably one resembilng the Bacillus Bifidus, and the corresponding diminution of the B. Coli Communis; marked restlessness at night; the disturbance of the circulation associated with cold hands and feet, and clammy perspiration especially affecting the skin of the scalp, face and neck; and lastly, inertia, causing the child to be content to sit quietly all day long rather than engage in the ordinary activities of childhood.

The retarding of growth he showed very conclusively to be due to the excessive elimination of lime salts, principally in the form of soaps of the fatty acids, elimination taking place through the feces. By a series of carefully worked out experiments he showed that the amount of lime eliminated in this way would account for the failure to add the normal increment to the child's weight in the twelve months. This loss of lime was greater after the ingestion of certain starches and fats, than when the patient was on another diet. The restlessness, the disorders of the circulation, and inertia might be accounted for as in rickets, or in any other disease of malnutrition. These conditions improve or grow worse with the amount of indican in the urine, which, in turn, increases according to the amount of the proteids in the diet of such form as to furnish substances capable of putrefaction.

The most marked and characteristic symptom is the presence of certain Gram positive bacteria in large numbers, and the corresponding diminution of the colon bacilli. With the disappearance of the B. Bifidus, and the increase of the colon B., all of the above symptoms improve. Dr. Herter has reported nine cases of this condition, all of which have been most carefully studied. Within the last few months I have come in contact with three of these cases, and the object of this paper is to call attention to the very excellent work done by Dr. Herter, and to give a brief report of these three cases, to date. The last one, however, having been under treatment such a brief time, the report will include merely a statement of it.

I regret that I have been unable to carry out many of the careful analyses made by Dr. Herter in the elimination of the lime salts excreted in the feces, but have not had at my disposal the means of doing this. I shall give only the salient features in the reports of these cases, and not weary you with details of treatment and conditions day by day.

Case 1, J. W., female, age five, child of well-to-do parents. Family history is negative; father and mother both healthy, one brother died of intussusseption, one younger sister is living and well. This child was breast fed, and perfectly normal up to 18 months of age. During her second summer she weighed 25 lbs. when she had an attack of entero-colitis. This lasted practically all the summer. The patient spent practically all the summer in the mountains, and returned with beginning rickets, and at that time weighed only 17 lbs. During that winter was taken from the breast—much to the disapproval of the mother, who had been urged to wean the child at twelve months and put on normal diet for a child of that age, the principal article being milk. The patient improved, and the end of the winter weighed about 30 lbs. During the next summer she began to have attacks of biliousness, so called, and the mother began to notice as she had noticed before during the winter, that the patient was not able to digest certain starches—notably potatoes. During the following summer patient was quite well, and the succeeding winter had a number of attacks of bronchitis, and in the late winter a severe attack of measles. During that whole twelve months she had gained but little. In June she was sent to the mountains, where she seemed to improve for the first month until a severe attack of auto-intoxication lasting 10 days decided her mother to return to the city. This was about the 15th of August. We had noticed her restlessness at night, her languor, her cold, clammy perspiration, cold hands and feet, sweating about the head and neck, her failure to grow normally, and the frequency of these attacks of intoxication. The works of Dr. Herter had come to my attention, and the case seemed to be so typical, that I made an examination of the urine and stools to see if the conditions there corresponded with those he had described. There were present in the stools a large number of Gram positive bacteria—notably the B. Bifidus, and there were very few colon bacilli. The urine was loaded with indican. I concluded that the diagnosis was that of acute infantilism as above described, and placed the patient on treatment, the details of which were Lactone buttermilk, rice, Huntley & Palmer biscuit, fruit juices, and gelatine, with occasionally broiled beef and lamb chops. Under this treatment she has gained up to 39 lbs., the normal for her age being about 41 lbs. The indican has disappeared from her urine, and the type of the intestinal flora has changed. The least indiscretion in diet, giving her too much starch, will bring about a recurrence of indicanuria, will increase the number of B. Bifidus, Gram positive bacteria, and will give foul grayish stools containing an excessive quantity of fats. The outlook for this case is good.

The most of this child's gain was made up until the last of November. At that time her father insisted on giving her some cake at a party, which resulted in relapse, and the loss of all of her gain. But now she has finally passed her former weight—weighing 40½ lbs. Her people realize the importance of a careful diet, and I anticipate a marked improvement. The treatment of her case has been almost entirely dietetic, the only medicines having been given her have been lime salts in the form of hypophosphites, and doses of calomel as needed.

Case 2, F. J., age 20 months, of good parentage.

The family history in this case is negative. He is the second child, of healthy parents, who have no constitutional troubles on either side. His older brother is a perfectly normal child. He grew normally until the age of four months. The only thing in his family history that might account in some measure for at least a part of his trouble is the fact that at six weeks his mother was threatened with an abortion, but, after a week's time, had no further trouble. When this child was born he weighed 8 pounds. At five months of age he weighed 15 pounds. At that time the physician who was treating him discovered a distinct murmur indicating pulmonary stenosis. Noticing that he did not gain in weight, he was put on cow's milk, and had a severe attack of entero-colitis. This was in the summer of 1908. He was never able apparently to digest cow's milk, and was finally fed on malted milk, beef juice, condensed milk, and later on quantities of toast, crackers and eggs, and orange juice. During the early winter of 1908-09 he had an attack of bronchitis, to which he was quite subject, which in April, 1909, developed into bronchial pneumonia. I went to his home in October to see the case, and besides receiving the above history, was told by the mother that the patient had not gained anything in weight for the whole year—weighing at that time 14½ pounds. He was exceedingly restless at night, was subject to frequent and profuse perspirations about the head and neck, was constipated, occasionally having grayish, foul smelling stools with diarrhœa. Was very much emaciated, anæmic, with a more or less rhachitic head, enormously distended abdomen, had no teeth, skin was dry and flabby. Examination of chest showed enlarged heart, with a very loud pulmonary murmur, lungs normal, liver normal, spleen normal, and abdomen normal, except for the distension. The blood count showed reds and whites normal, hæmoglobin 60 per cent., the stools containing large quantities of Gram positive bacteria, and excessive quantities of fat. The urine contained a very large quantity of indican. At the time that I saw him the diet consisted largely of starches in the form of condensed milk, toast, mashed potatoes with eggs, and beef juice. The lack of growth and his whole condition had been attributed to the heart lesion. He had no sympttoms however, of lack of compensation, his pulse was normal, and I made a diagnosis of acute infantilism.

About two weeks later his family moved to Atlanta—his father having changed his business—and I have had the child under observation ever since. For the whole of October he had an intense diarrhœa with mucous stools, undigested, with all the appearances of colitis. He lost about two pounds during that time. I persisted, however, in the diet I had been giving him—consisting at that time of beef juice, gelatine in large quantities, rice gruel, and liquid peptonoids. The ordinary measures were used for correcting the entero-colitis. By the end of October he had cut two teeth. Since that time he has cut six more. As soon as the diarrhœa was corrected, I began giving sweet milk diluted with arrow root and gelatine—beginning first with one dram of sweet milk, and in a 6-ounce mixture, gradually increasing until now he is taking whole milk with one dram of arrow root in 8 ounces of milk. He has had from one to six Huntley & Palmer biscuits, a bowl of soft rice, one egg, and one scraped beef ball a day. He has gained steadily, until now he weighs nearly 21 pounds; is talking, and walking everywhere; his abdomen has gone down to normal size, and his hæmoglobin has increased to normal percentage. His movements are normal, having two a day, well digested. They show no trace of B. Bifidus. The urine is free from indican. He has had one or two slight attacks of bronchitis, which he has thrown off in a few days, and except for the fact that his nights are not as quiet as they should be, he is apparently a perfectly well child. Of course, his weight is not up to the average weight of a child of his age as yet, but with the steady gain that he has shown, it seems that it will only be a question of a short time until he will have attained that.

Case 3, O. H., female child of good parents, age 5.

This patient has been under observation but for a brief time. Further than to state that she is underweight, has shown the presence of B. Bifidus in the stools, with frequent attacks of auto-intoxication, and has recently been eating large quantities of starch, I have nothing to report as yet.

As stated above, I merely desired to call attention to this condition, as I am sure that there are other cases occurring in the practice of some of the members of this Society. The outlook is good for these patients if a careful diet is followed. We all come in contact with many cases of under-development, and I believe that at least some of these from a careful study will be found to have as the underlying causes, this intestinal condition, which can be corrected, and which can insure to these little fellows a normal growth and physical development, which, at least, is their right.

He is now taking lactone buttermilk instead of sweetmilk, is bright, has all his teeth, and weighs 22 lbs.

